# Coping With the Impact of COVID-19 Safety Recommendations: The Importance of Pets

**DOI:** 10.1093/geroni/igaa057.3432

**Published:** 2020-12-16

**Authors:** Kerstin Emerson, Deborah Kim, George Mois, Jenay Beer

**Affiliations:** 1 University of Georgia, Athens, Georgia, United States; 2 University of Georgia, Lawrenceville, Georgia, United States; 3 Institute of Gerontology, Athens, Georgia, United States

## Abstract

The COVID-19 pandemic is disproportionately affecting older adults. Initial research suggests that older adults are experiencing negative emotions and stress during social distancing and self-quarantining. Consistently, literature shows that pet ownership can mitigate some negative emotions, including stress and loneliness. Our goal was to explore the impact of pets on older adults who were restricting their social interactions during the first three to five weeks of the COVID-19 pandemic. The study was based on web-based survey data from 833 persons aged 60 and older in the United States. The sample age of respondents ranged from 60-85, with a majority (80.2%) female and White (95%). About 56% of the sample reported that they owned at least one pet. Respondents with pets did not significantly differ in stress or loneliness measures compared to those without pets. A majority of respondents reported engaging in more distance communication compared to before the pandemic. However, respondents with pets were less likely to report an increase in the following technology-mediated forms of communication: emails, texts, computers, and telephone (all p-values < .05) compared to those without pets. There was no significant difference in other coping mechanisms (e.g. exercise, alcohol intake) by pet ownership. These results suggest that respondents did not rely on pets to reduce stress or loneliness. However, pets may fill other communication/connection needs, and older adults may then rely less on communication with other people. Research on coping with the impact of Covid-19 has implications for interventions, including specific interventions for pet ownership.

